# Diabetes Worsens Skeletal Muscle Mitochondrial Function, Oxidative Stress, and Apoptosis After Lower-Limb Ischemia-Reperfusion: Implication of the RISK and SAFE Pathways?

**DOI:** 10.3389/fphys.2018.00579

**Published:** 2018-05-22

**Authors:** Julien Pottecher, Chris Adamopoulos, Anne Lejay, Jamal Bouitbir, Anne-Laure Charles, Alain Meyer, Mervyn Singer, Valerie Wolff, Pierre Diemunsch, Gilles Laverny, Daniel Metzger, Bernard Geny

**Affiliations:** ^1^Fédération de Médecine Translationnelle de Strasbourg, Faculté de Médecine, Institut de Physiologie, Equipe d'Accueil EA3072 “Mitochondrie, Stress Oxydant et Protection Musculaire”, Université de Strasbourg, Strasbourg, France; ^2^Pôle Anesthésie Réanimations Chirurgicales SAMU/SMUR (POLARS), Hôpital de Hautepierre, Service d'Anesthésie-Réanimation Chirurgicale, Hôpitaux Universitaires de Strasbourg, Strasbourg, France; ^3^Department of Cardiology, St. Paul General Hospital, Thessaloniki, Greece; ^4^Service de Chirurgie Vasculaire, Hôpitaux Universitaires de Strasbourg, Strasbourg, France; ^5^Service de Physiologie et d'Explorations Fonctionnelles, Hôpitaux Universitaires de Strasbourg, Strasbourg, France; ^6^Bloomsbury Institute of Intensive Care Medicine, University College London, London, United Kingdom; ^7^Unité Neurovasculaire, Hôpitaux Universitaires de Strasbourg, Strasbourg, France; ^8^Centre National de la Recherche Scientifique, UMR7104, Institut National de la Santé et de la Recherche Médicale U1258, Institut de Génétique et de Biologie Moléculaire et Cellulaire, Université de Strasbourg, Illkirch, France

**Keywords:** diabetes, ischemia-reperfusion, peripheral arterial disease, protective kinases, muscles, mitochondria

## Abstract

**Objectives:** Diabetic patients respond poorly to revascularization for peripheral arterial disease (PAD) but the underlying mechanisms are not well understood. We aimed to determine whether diabetes worsens ischemia-reperfusion (IR)-induced muscle dysfunction and the involvement of endogenous protective kinases in this process.

**Materials and Methods:** Streptozotocin-induced diabetic and non-diabetic rats were randomized to control or to IR injury (3 h of aortic cross-clamping and 2 h of reperfusion). Mitochondrial respiration, reactive oxygen species (ROS) production, protein levels of superoxide dismutase (SOD2) and endogenous protective kinases (RISK and SAFE pathways) were investigated in rat gastrocnemius, together with upstream (GSK-3β) and downstream (cleaved caspase-3) effectors of apoptosis.

**Results:** Although already impaired when compared to non-diabetic controls at baseline, the decline in mitochondrial respiration after IR was more severe in diabetic rats. In diabetic animals, IR-triggered oxidative stress (increased ROS production and reduced SOD2 levels) and effectors of apoptosis (reduced GSK-3β inactivation and higher cleaved caspase-3 levels) were increased to a higher level than in the non-diabetics. IR had no effect on the RISK pathway in non-diabetics and diabetic rats, but increased STAT 3 only in the latter.

**Conclusion:** Type 1 diabetes worsens IR-induced skeletal muscle injury, endogenous protective pathways not being efficiently stimulated.

## Introduction

Diabetes is a major risk factor for peripheral arterial disease (PAD) and in patients with diabetes, PAD is more severe and has a poorer response to revascularization (Jude et al., 2001; DeRubertis et al., 2008; Malmstedt et al., 2008). Yet, the mechanisms linking diabetes to worse outcomes in PAD are not well understood. Diabetes *per se* impairs skeletal muscle mitochondrial function (Kelley et al., 2002; Bonnard et al., 2008; Anderson et al., 2009). In type 1 diabetic patients the skeletal muscles present reduced mitochondrial oxidative phosphorylation, even without obvious vascular abnormalities (Karakelides et al., 2007) and the mitochondrial impairment may even precede hyperglycemia in type 2 diabetes (Petersen et al., 2004). These data suggest that mitochondrial dysfunction may be a common pathway of diabetes and PAD severity.

When PAD ischemia becomes critical, blood flow has to be reestablished by revascularization. However, the return in blood flow causes additional muscle damage. This paradoxical and detrimental effect is known as ischemia-reperfusion injury (IRI). Although the cause of PAD is occlusive arterial disease, muscle mitochondrial dysfunction is also critical for the severity of PAD. These abnormalities include impaired mitochondrial respiration and increased oxidative stress, and are present in both chronic PAD and after ischemia-reperfusion (IR) (Pipinos et al., 2006; Makris et al., 2007; Tran et al., 2012; Guillot et al., 2014; Lejay et al., 2014, 2017; Paradis et al., 2016). While it is generally assumed that muscle mitochondria are more susceptible to IRI in diabetic subjects, data supporting this assertion are lacking. Very little is known regarding the magnitude of the mitochondrial dysfunction and the mechanisms involved in this process.

It is well established that myocardial IR induces the reperfusion injury salvage kinase (RISK) and survivor activating factor enhancement (SAFE) protective pathways (Lecour, 2009; Rossello and Yellon, 2017). Decreased myocardial activation of RISK and SAFE increases the susceptibility of the diabetic heart to IRI (Tsang et al., 2005; Drenger et al., 2011). Acute activation of RISK and SAFE effectors appear to converge on the mitochondria and avert cell damage. In brief, RISK signaling involves protein kinase B (Akt) phosphorylation which, in turn, phosphorylates and inactivates glycogen synthase kinase 3β (GSK-3β). Inactivated GSK-3β cannot induce mitochondrial permeability transition pore (mPTP) opening anymore, thus preventing mitochondrial dysfunction and apoptosis (Juhaszova et al., 2004). SAFE activation involves phosphorylation and hence, activation of signal transducer and activator of transcription 3 (STAT3). Activated STAT3 stimulates mitochondrial respiration, inhibits mPTP opening and attenuates apoptosis. Yet, the involvement of these pathways in skeletal muscle IR is unknown.

The purpose of this study was to investigate the impact of type 1 diabetes on the skeletal muscle IRI and examine the activation of the RISK and SAFE pathways. We assessed mitochondrial respiration, oxidative stress, and effectors of apoptosis and of RISK and SAFE pathways, in streptozotocin-treated, type 1 diabetic rats, in comparison to non-diabetic controls.

## Materials and methods

### Experimental animals

Experiments were performed on 8-week-old male Wistar rats (Depré, Saint-Doulchard, France), either vehicle-treated or streptozotocin-treated to induce insulin-dependent type 1 diabetes. The study conformed to the “Principles of laboratory animal care” (NIH publication 85–23, revised 1985) and was approved by the Institutional Animal Care Committee (CREMEAS AL/02/10/06/2009).

### Induction of diabetes

Type 1 diabetes was induced in male rats by a single 65 mg/kg streptozotocin injection in the penile vein. Animals were considered diabetic when blood glucose was above 16.7 mmol/L, 8 days after induction of diabetes. Non-diabetic vehicle-treated animals received intravenous saline injection at the same time. Six weeks after diabetes induction, streptozotocin-treated and vehicle-treated rats were individually housed in metabolic cages for 2 days. After the first day (considered an acclimation period), blood glucose concentrations, food and water intake and urine output were recorded over a 24-h period.

Thirty days after diabetes induction, an oral glucose-tolerance test was performed in diabetic rats and non-diabetic animals to confirm the diabetic status of the formers. The test consisted in a 2 g/kg glucose loading given by oral gavage after a 12-h fasting period. Glucose concentrations were determined on tail vein blood at 20, 40, 60, 120, and 180 min after gavage.

### Animal surgical preparation and procedure

As previously described (Mansour et al., 2012), after performing a midline laparotomy under isoflurane anesthesia, the infra-renal abdominal aorta was dissected and freed from adjacent adhesions. All arterial collaterals located between the renal arteries and the aortic bifurcation, were coagulated and sectioned using electrocautery (Geiger®, thermal cautery unit).

### Experimental design

Seven weeks after vehicle or streptozotocin injection, rats (referred to as non-diabetic “n,” and diabetic “d,” respectively) were randomly assigned to the control (CON) or IR group. The control groups (nCON, dCON, 8 rats per group) underwent 5 h of isoflurane anesthesia and similar surgical manipulation to the IR groups, except for hindlimb ischemia (sham-operated). The ischemia–reperfusion groups (nIR and dIR, 10 rats per group) underwent 3 h of ischemia induced by infra-renal aortic occlusion and collateral vessel ligation, followed by 2 h of reperfusion. Ischemia was clinically characterized by cyanosis and lack of an arterial pulse distal to the clamp, and biochemically by an increase in capillary blood lactate measured in the right hindlimb (Lactate Pro device, LT1710; Arkray, KGK, Japan).

After reperfusion, gastrocnemius muscles, that are considered more sensitive to IR (Charles et al., 2017), were harvested and either analyzed immediately (mitochondrial respiration) or kept in ice or in liquid nitrogen-cooled isopentane. Animals were sacrificed by heart retrieval under deep anesthesia (5% isoflurane).

### Study of muscle mitochondrial respiration in skinned fibers

Mitochondrial respiration was studied in saponin-skinned fibers of white gastrocnemius muscle (glycolytic muscle), as previously described (Talha et al., 2013). Fibers were separated and subsequently permeabilized in a bath of solution S containing 50 μg/ml saponin for 30 min at 4°C, under shaking. Permeabilized fibers were washed for 10 min under shaking, to remove the saponin, and placed in a bath with the respiratory solution for 5 min twice, in order to remove any phosphates. Finally, oxygen consumption was measured polarographically with a Clark-type electrode in a 3 ml oxygraphic cell (Strathkelvin Instruments, Glasgow, Scotland) at 22.1°C in incubation buffer using 5 mM glutamate and 2.5 mM malate as substrates for complex I, or 25 mM succinate (in combination with 0.02 mM amytal to inhibit complex I) as substrate for complex II.

After recording of basal oxygen consumption (V_0_), maximal fiber respiration (V_Max_) rate was measured under continuous stirring in the presence of a saturating amount of ADP (2 mM) as a phosphate acceptor. Relative contributions of the respiratory chain complexes I, III and IV to the global mitochondrial respiratory rates were also determined. When V_Max_ was recorded, the electron flow went through complexes I, III, and IV. For determining V_Succ_, complex I was blocked with amytal (0.02 mM) and complex II was stimulated with succinate (25 mM). Mitochondrial respiration in these conditions allowed to determine the contribution of complexes II, III, IV activities. Thereafter, N, N, N′, N′-tetramethyl-p-phenylenediamine dihydrochloride (TMPD, 0.5 mM) and ascorbate (0.5 mM) were added as an artificial electron donor to cytochrome c. In these conditions, the activity of cytochrome c oxidase (complex IV) was determined as an isolated step of the respiratory chain (V_TMPD_). In all cases, mitochondrial respiration assays in skinned fibers were performed immediately after harvesting. Fibers were then dried for 15 min at 150°C and respiration rates were expressed as μM O_2_/min/g dry weight.

### Assessment of oxidative stress in skeletal muscles

#### Assessment of reactive oxygen species production in skeletal muscle by dihydroethidium staining

As described previously (Pottecher et al., 2013), 10 μm-thick serial sections of white gastrocnemius muscle were prepared using a cryostat microtome and incubated with dihydroethidium (DHE) that produces red fluorescence when oxidized to ethidium bromide (EtBr) by ROS, including superoxide anion (Li and Jackson, 2002; Sheetz and King, 2002; Charles et al., 2011). To assess ROS production in each skeletal muscle section, mean fluorescence intensity (arbitrary units) was determined in 25 regions of interest under 20× epifluorescence magnification.

As ROS production by diabetic skeletal muscle mitochondria may be confounded by concurrent changes in respiration rates, this may leave raw, unadjusted ROS levels unchanged, while specific ROS production may increase, when considered relative to electron transport (Herlein et al., 2011). Consequently, the mean DHE fluorescence was divided by the mitochondrial respiration rate from skinned fibers.

#### Protein levels of the antioxidant mitochondrial manganese superoxide dismutase in skeletal muscles by western blot

This assessment is detailed below in the following paragraph.

### Protein extraction and analysis

Gastrocnemius muscles were grounded in a mortar at 4°C in RIPA buffer [50 mM Tris pH 7.5, 1% Nonident P40, 0.5% sodium deoxycholate, 0.1% SDS, 150 mM NaCl, 5 mM EDTA, 1 mM PMSF and phosphatase and protease inhibitor cocktails according to the manufacturer's protocol (PhosphoStop and Complete-Mini EDTA free, Roche)]. Homogenates (50 μg of protein, *n* = 3–6 per group) were electrophoresed on polyacrylamide gels. Proteins were electroblotted onto nitrocellulose membranes using a Trans-blot turbo transfer system (Biorad) and immunodetected using primary antibodies directed against SOD2 (SOD-110, StressGen), Akt (4691, Cell Signaling), phospho-Akt Ser473 (4060, Cell Signaling), phospho-Akt Thr308 (4056, Cell Signaling), GSK-3β (610201, BD Biosciences), phospho-GSK-3β Ser9 (5558, Cell Signaling), STAT3 (9132, Cell Signaling), phospho-STAT3 Tyr705 (9131, Cell Signaling), cleaved caspase-3 (9661, Cell Signaling), tubulin and GAPDH (MAB374, Millipore Upstate Chemicon), according the manufacturer's instructions. Proteins were revealed with secondary antibodies conjugated to horseradish peroxidase (Amersham Biosciences) using an enhanced chemiluminescence detection system (ECLplus, GE Healthcare) and an ImageQuant™ LAS 4000 biomolecular imager (GE Healthcare). Immunodetected proteins were quantified with the FIJI software and normalized to GAPDH levels (except cleaved caspase-3, which was normalized to tubulin levels).

### Statistics

All data are expressed as mean ± standard error of the mean (SEM), and were analyzed using Prism software (GraphPad Prism 5, Graph Pad Software, San Diego, USA). Comparisons between two groups were performed by Student two-tailed *t-*test. Two-way analysis of variance (ANOVA) was applied to test simultaneously the main effects and interaction for diabetes status and ischemia. Comparisons between more than two groups were performed using one-way ANOVA with Newman-Keuls *post-hoc* correction for multiple comparisons. Repeated-measures ANOVA was conducted when appropriate. In all cases, a *p*-value < 0.05 was considered statistically significant.

## Results

### Hemodynamic and metabolic changes in diabetic and non-diabetic animals

**Figure 1:**
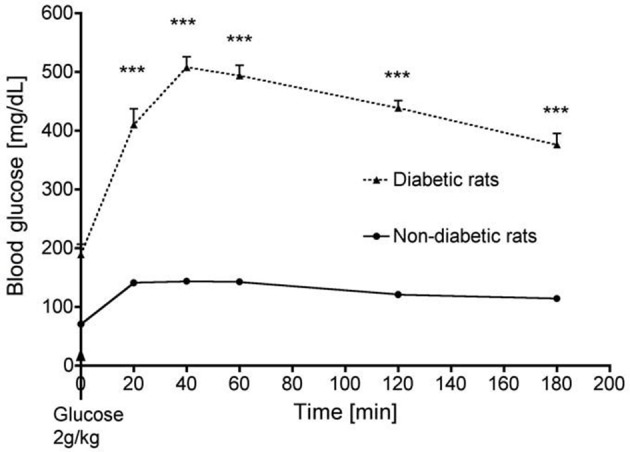
Time course of blood glucose in streptozotocin-treated (diabetic) and non-diabetic rats during an oral glucose tolerance test. ***p < 0.001.

Compared to vehicle-treated animals, streptozotocin-treated rats displayed typical features of type 1 diabetes with hyperglycemia, polyuria, polydipsia, polyphagia and decreased body weight. Baseline heart rate was similar in both groups although streptozotocin-treated rats exhibited mild arterial hypertension (164 ± 5, mmHg vs. 140 ± 3; *p* < 0.001). The absolute and relative maximal increases in blood glucose were higher in streptozotocin-treated than in the non-diabetic rats (+319 ± 9 mg/dL vs. +73 ± 4 mg/dL and 309 ± 24% vs. 200 ± 4%, respectively; *p* < 0.001) (Figure 1). Hind limb capillary blood lactate levels pre-IR were similar between streptozotocin- and vehicle-treated animals (2.8 ± 0.3 and 2.6 ± 0.4 mmol/L respectively, *p* = NS), and were similarly increased at the end of the 3 h ischemia (17.7 ± 0.5 vs. 18.9 ± 0.9 mmol/L respectively, *p* = NS).

Heart rate changes were also not statistically different between diabetic and non- diabetic animals at baseline, at the end of ischemia and after 2 h of reperfusion (data not shown). These results suggest that streptozotocin- and vehicle-treated animals were subjected to a similar ischemic insult.

### Mitochondrial respiratory chain complex activities are more impaired after IR in diabetic animals than in controls

**Figure 2:**
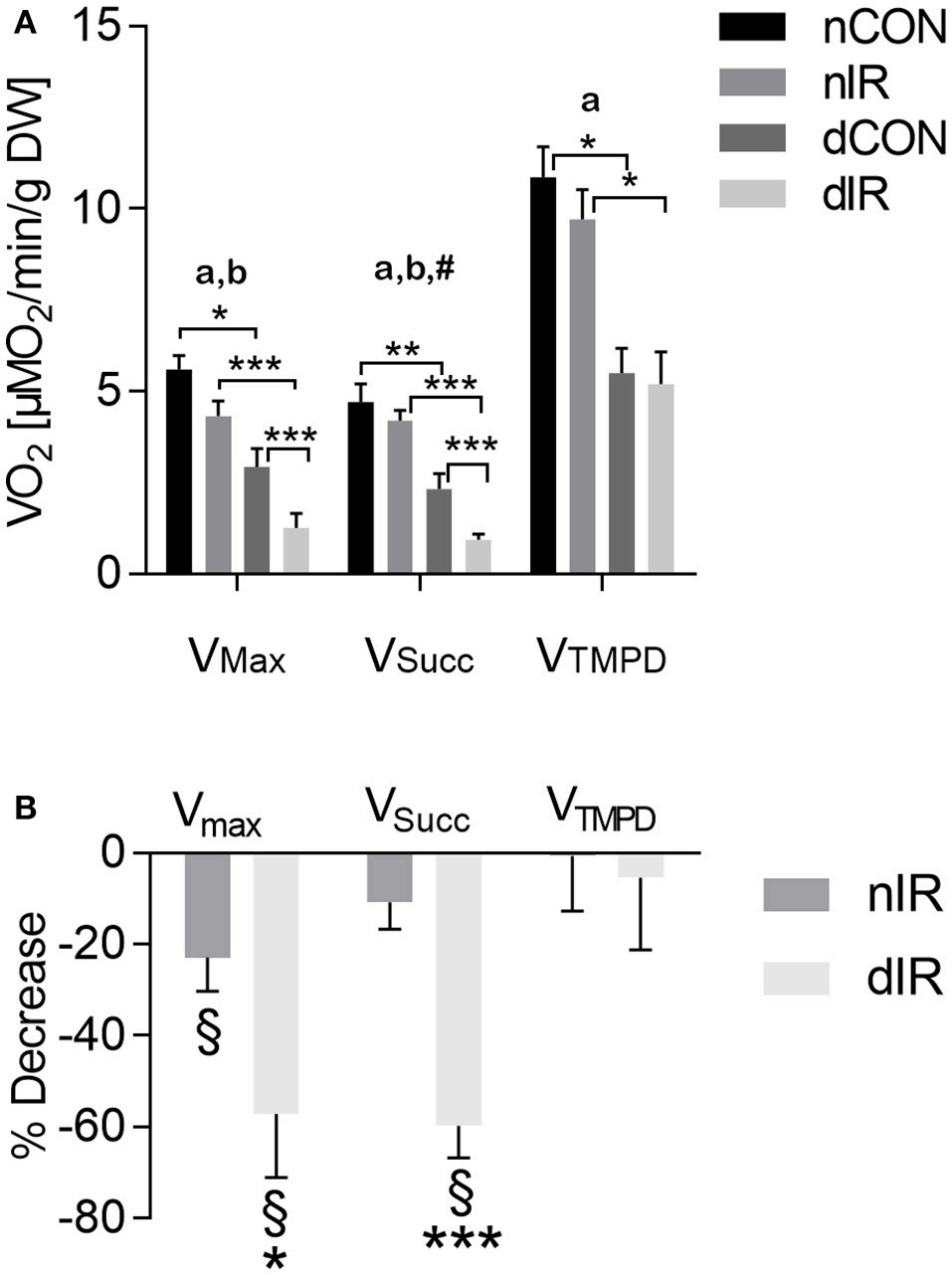
Mitochondrial respiration in gastrocnemius muscles from vehicle-treated and streptozotocin-treated rats with and without hindlimb ischemia-reperfusion. **(A)** Mitochondrial respiration was determined in gastrocnemius muscles from sham-operated, vehicle-treated (nCON) or streptozotocin-treated rats (dCON), and after ischemia reperfusion in vehicle-treated (nIR) and streptozotocin-treated rats (dIR). Maximal fiber respiration (V_Max_) rate, combined complexes II, III, IV activities (V_Succ_) and isolated complex IV activity (VTMPD) were measured. Results are expressed as mean ± SEM [μM O_2_/min/g dry weight tissue]. *p < 0.05; **p < 0.01; ***p < 0.001, ^a^*p* < 0.05 for diabetes effect; ^b^*p* < 0.05 for IR effect; ^#^*p* < 0.05 for interaction. **(B)** Variations in mitochondrial respiration in gastrocnemius muscles obtained after ischemia-reperfusion vs. sham operation in vehicle-treated (“nIR”) and streptozotocin- treated rats (“dIR”). Results show a variation in oxygen consumption after ischemia- reperfusion vs. sham operation, expressed as percent decrease ± SEM. ^§^indicates that the variation is significantly different from zero, while *p < 0.05 and ***p < 0.001 indicate that the variation is significantly different between diabetic and non-diabetic rats.

Among controls, oxygen consumption was halved in streptozotocin-treated rats as compared to non-diabetic rats (Figure 2A). Ischemia-reperfusion decreased V_Max_ in both diabetic and non-diabetic animals (Figure 2A). However, V_Max_ was significantly more reduced after IR in type 1 diabetic rats compared to the non-diabetics (−57 ± 14% vs. −23 ± 7%; *p* < 0.05, Figure 2B). While IR did not alter V_Succ_ in non-diabetic animals (−11 ± 6%; *p* = NS), it induced a significant decrease in V_Succ_ in diabetic rats (−60 ± 7%; *p* < 0.001 compared to non-diabetic animals). IR did neither alter V_TMPD/Asc_ in streptozotocin- nor in non-diabetic animals (Figure 2B). These results show that, after IR, mitochondrial respiration is more impaired in diabetic than in non-diabetic animals.

### Larger increase in oxidative stress in diabetic animals

**Figure 3:**
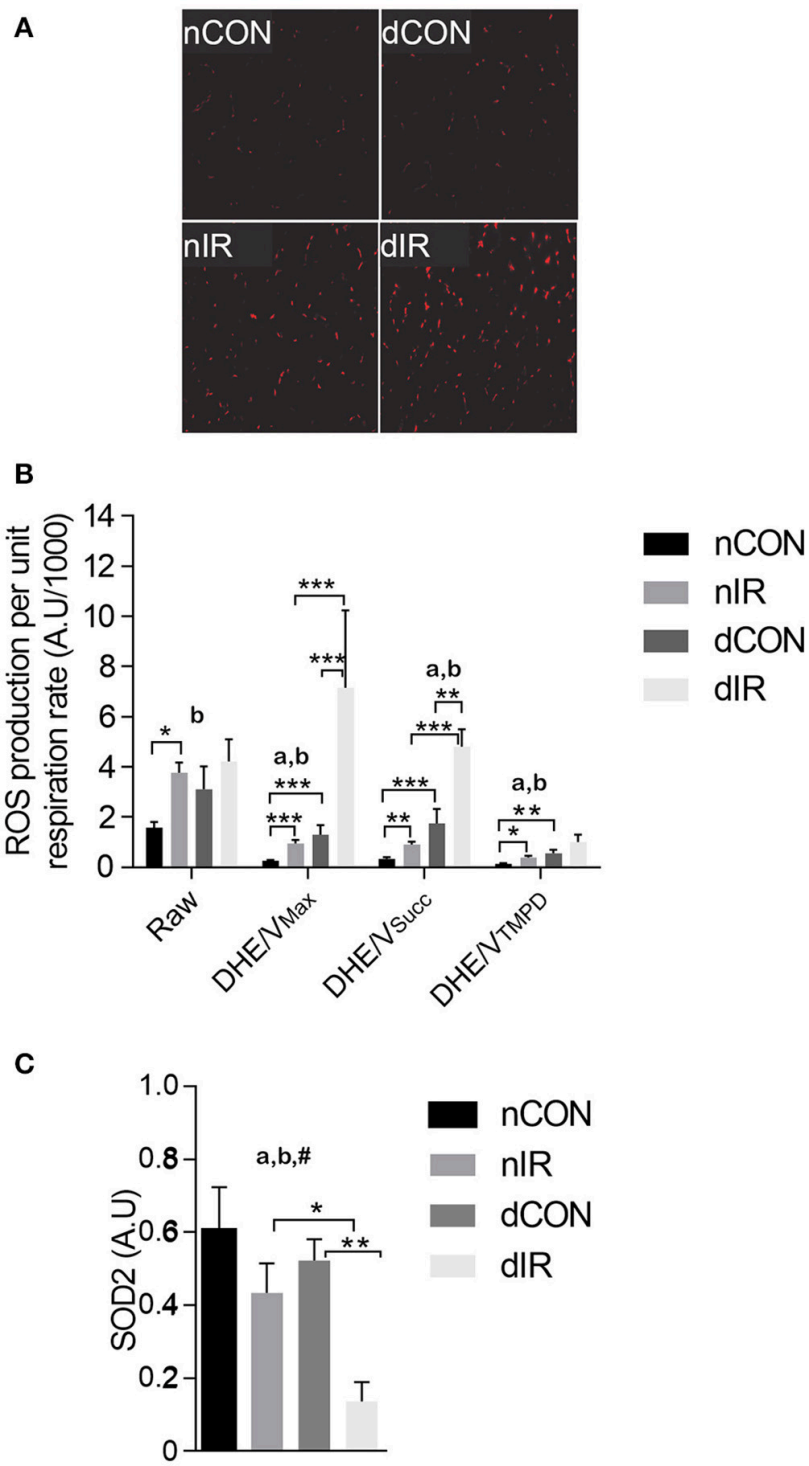
Reactive oxygen species production and antioxidant protein level. **(A)** Representative histological sections after dihydroethidium (DHE) staining in sham- operated, vehicle-treated (nCON) or streptozotocin-treated rats (dCON), and after ischemia reperfusion in vehicle-treated (nIR) and streptozotocin-treated rats (dIR). **(B)** Raw and specific (normalized to respiration rate) reactive oxygen species (ROS) production in gastrocnemius muscles from sham-operated, vehicle-treated (nCON) or streptozotocin-treated rats (dCON), and after ischemia reperfusion in vehicle-treated (nIR) and streptozotocin-treated rats (dIR). Data are expressed as mean ± SEM [Arbitrary Unit/μM O_2_/min/g dry weight]. *p < 0.05; **p < 0.01; ***p < 0.001, ^a^*p* < 0.05 for diabetes effect; ^b^*p* < 0.05 for IR effect. **(C)** Quantification of mitochondrial superoxide dismutase (SOD2) protein level by Western blotting in gastrocnemius muscles from sham-operated, vehicle-treated (nCON) or streptozotocin-treated rats (dCON), and after ischemia reperfusion in vehicle-treated (nIR) and streptozotocin-treated rats (dIR). Data are expressed as the mean ± SEM of the intensity of the SOD2 bands reported to the intensity of the internal control (glyceraldehyde-3-phosphate dehydrogenase, GAPDH). *p < 0.05; **p < 0.01, ^a^*p* < 0.05 for diabetes effect; ^b^*p* < 0.05 for IR effect; ^#^*p* < 0.05 for interaction.

After IR, we observed a 2-fold increase in raw DHE fluorescence in vehicle-treated rats (*p* < 0.001). In contrast, DHE staining, which was already enhanced in the diabetic rats, was not further increased after IR in this group (Figures 3A,B). However, ROS levels normalized either to V_Max_ (DHE/V_Max_) or V_Succ_ (DHE/V_Succ_) were significantly increased after IR in both diabetic and non-diabetic rats, but to a larger extent in diabetic animals (Figure 3B).

The antioxidant SOD2 level was conversely 3-fold lower in diabetic rats after IR (Figure 3C). Thus, diabetic muscles produced more ROS and had reduced antioxidant defenses after IR.

### Safe and risk pathways implications in diabetic rats after IR

To test whether the alterations on mitochondrial function and oxidative stress after IR were associated with activation of the endogenous protective kinases, we investigated the effector proteins of RISK (Akt) and SAFE (STAT3) pathways.

**Figure 4:**
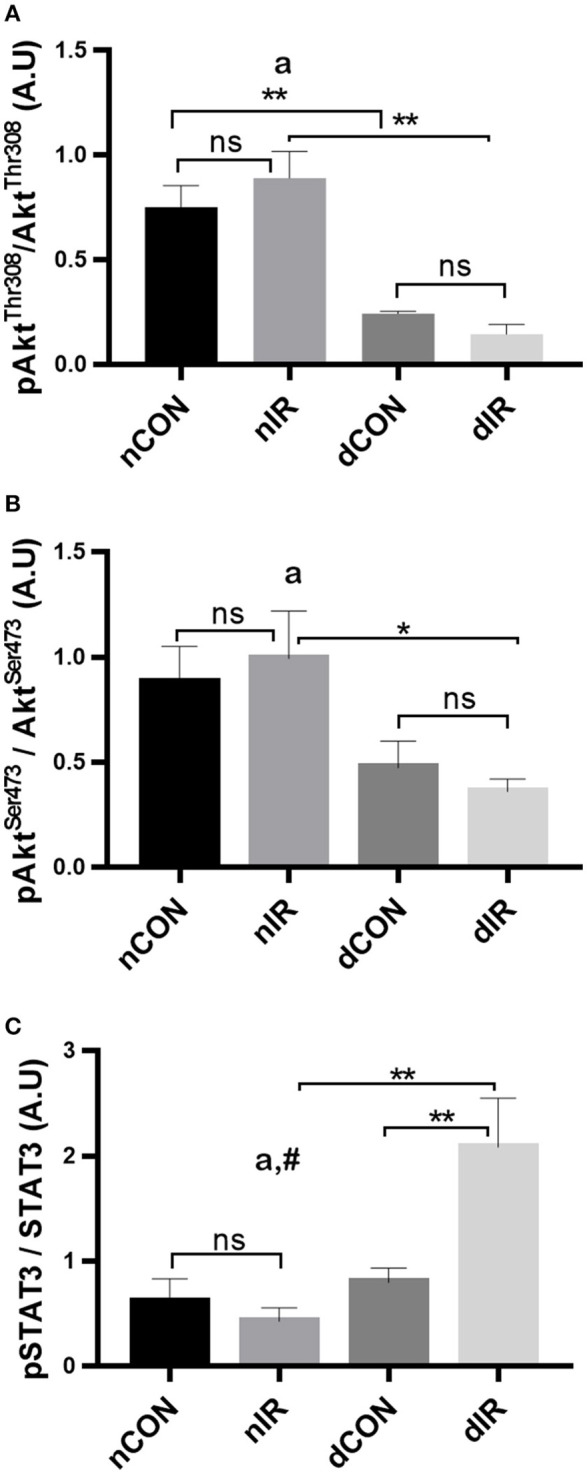
Protein levels of key components of the endogenous protective RISK and SAFE pathways. Quantification of the levels of effector proteins from the RISK pathway [Akt, phospho-Akt/Total Akt Thr308 **(A)**, phospho-Akt/Total Akt Ser473 **(B)**] and the SAFE pathway [STAT3, phospho-STAT3/Total STAT3 tyr705 **(C)**] in gastrocnemius muscle from sham-operated, vehicle-treated (nCON) or streptozotocin-treated rats (dCON), and after ischemia reperfusion in vehicle-treated (nIR) and streptozotocin-treated rats (dIR). GAPDH was used as an internal control for all proteins. Data are expressed as the mean ± SEM of the intensity of the bands reported to the intensity of the internal control. *p < 0.05; **p < 0.01, ^a^*p* < 0.05 for diabetes effect; ^#^*p* < 0.05 for interaction.

Among controls (no IR), the activated Akt (phosphorylated at the threonine 308 and the serine 473 residues) was higher in the non-diabetic rats (*p* < 0.01). After IR, the phosphorylated Akt levels did neither change in diabetic, nor in non-diabetic rats, indicating no RISK activation. However, both diabetic groups had consistently lower levels of activated Akt than their non-diabetic counterparts (*p* < 0.01; Figures 4A,B).

Regarding SAFE pathway, the active, phosphorylated form of STAT3 was not different between diabetic and non-diabetic rats who did not undergo IR. After IR, the phosphorylated STAT3 levels showed a significant 2-fold increase in the diabetic animals only (*p* < 0.01) (Figure 4C). Therefore, one effector of the SAFE pathway is activated by IR in diabetic but not in in the non-diabetic rats.

### Decreased GSK-3β inhibition and increased cleaved caspase-3 and cell damage in diabetic rats after IR

**Figure 5:**
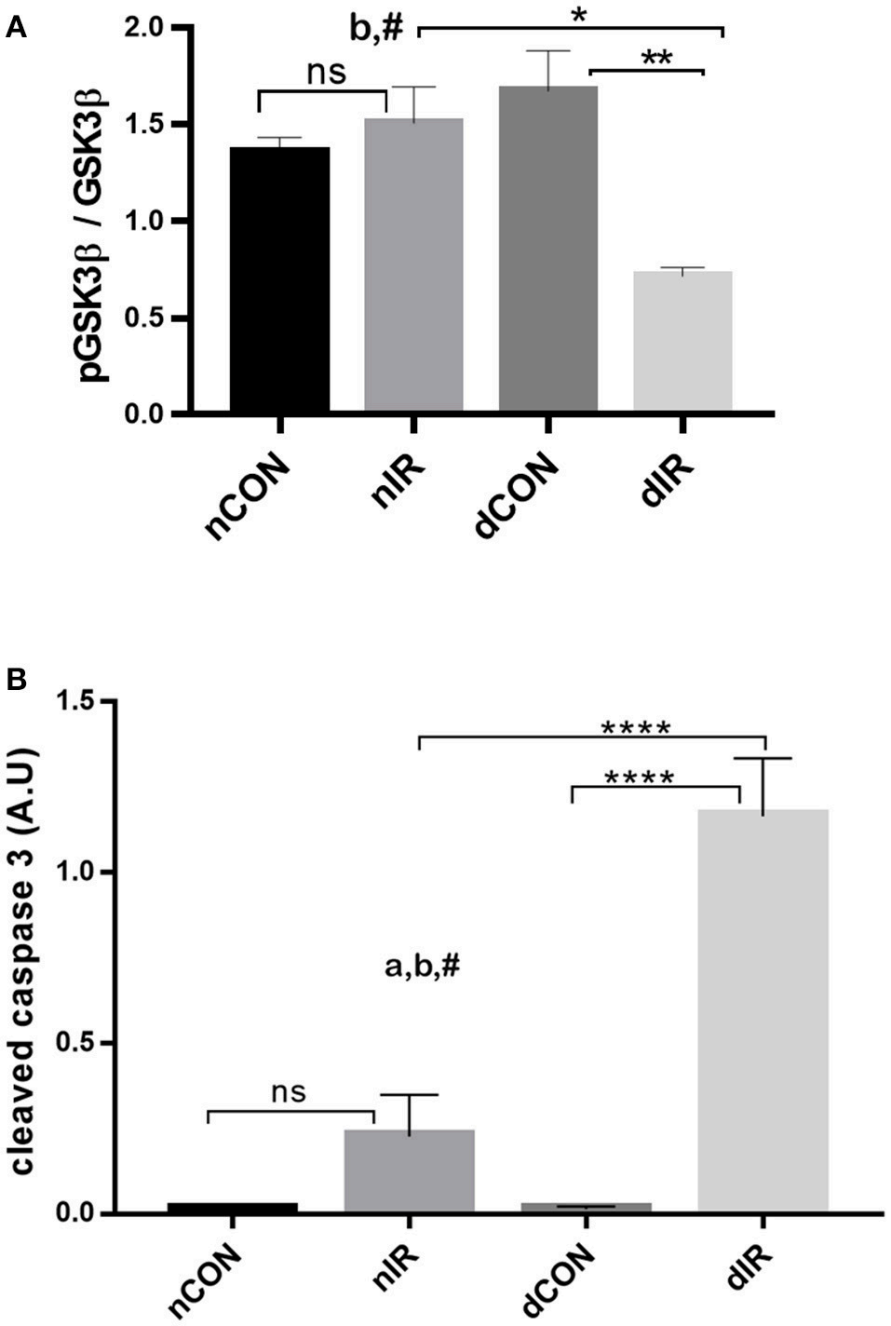
Protein levels of GSK-3β and cleaved caspase-3. Quantification of the levels of **(A)** GSK-3β, (GSK-3β, phospho-GSK-3β/Total GSK-3β Ser9) and **(B)** caspase-3 effector of apoptosis in gastrocnemius muscle from sham-operated, vehicle-treated (nCON) or streptozotocin-treated rats (dCON), and after ischemia reperfusion in vehicle-treated (nIR) and streptozotocin-treated rats (dIR). GAPDH was used as an internal control except cleaved caspase-3 for which tubulin was used. Data are expressed as the mean ± SEM of the intensity of the bands reported to the intensity of the internal control. *p < 0.05, **p < 0.001, ****p < 0.0001, ^a^*p* < 0.05 for diabetes effect; ^b^*p* < 0.05 for IR effect; ^#^*p* < 0.05 for interaction.

While the phosphorylated GSK-3β levels were not altered after IR in non-diabetic animals, they dropped markedly in diabetic rats (*p* < 0.01), resulting in a 2.5-fold decrease in phospho-GSK-3β/GSK-3β ratio (Figure 5A). Thus, after IR, the protective, phosphorylated GSK-3β was markedly lower in the diabetic than in the non-diabetic animals and was even lower compared to the diabetic rats that did not undergo IR (*p* < 0.05 and *p* < 0.01 respectively). Cleaved caspase-3 is a strong downstream effector of mitochondrial-mediated apoptosis and characterized as the “key executioner” of apoptosis.

IR was followed by an increased cleaved caspase-3 expression in gastrocnemius, which was non-significant in non-diabetic animals and highly significant in diabetic rats. Moreover, the increased expression of cleaved caspase 3 was 6-fold larger in the muscles of diabetic animals than in the muscles of their non-diabetic counterparts (*p* < 0.0001) (Figure 5B).

**Figure 6:**
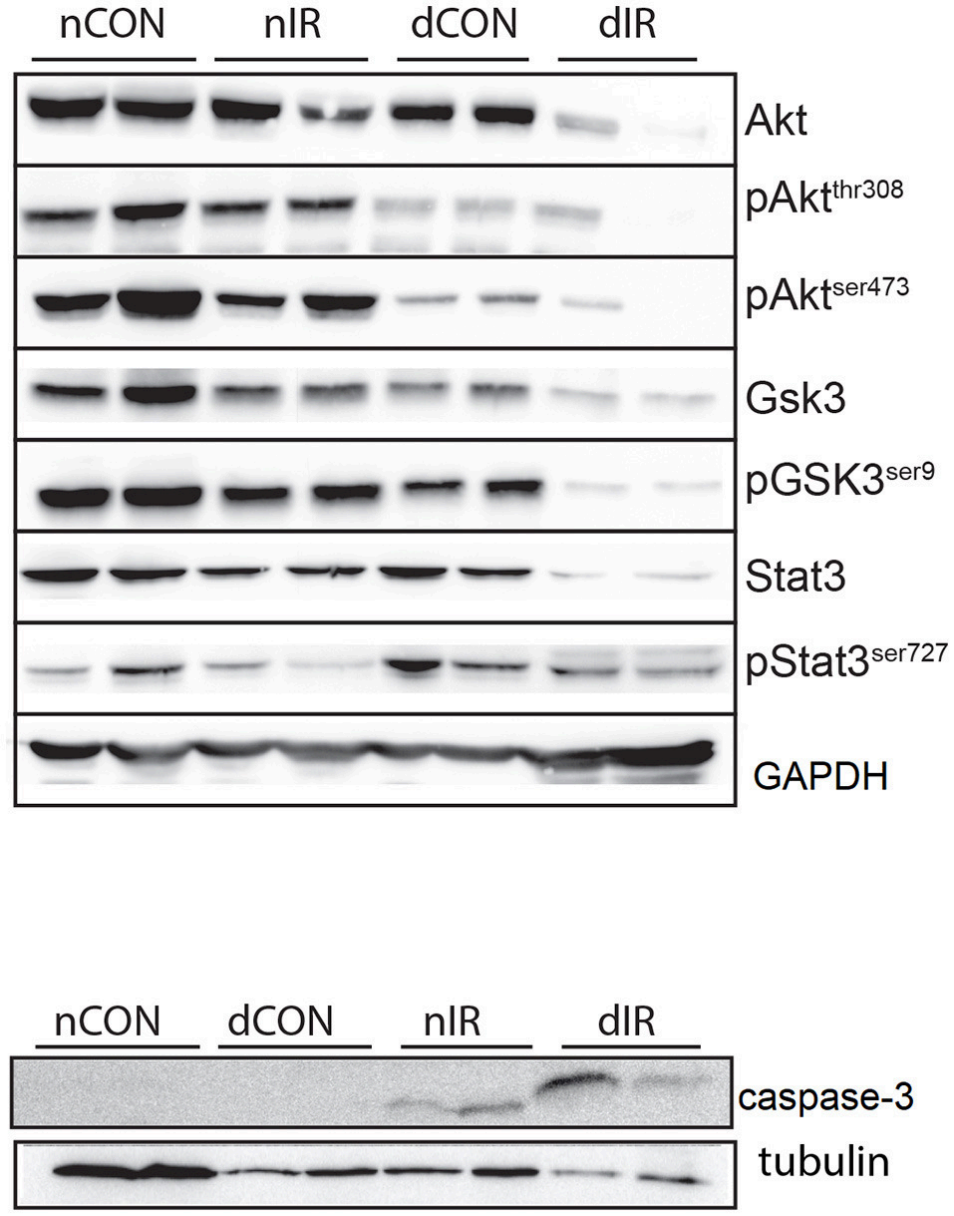
Representative Western blots of the effector proteins. **(Upper panel)** Effector proteins of RISK and SAFE pathways. **(Lower panel)** Cleaved caspase-3. GAPDH was used as an internal control except cleaved caspase-3 for which tubulin was used.

Taken together, the decreased levels of inactivated GSK-3β and the increased levels of cleaved caspase-3 in the diabetic animals after IR, denote a reduced protection against IRI and a strong trend for apoptosis (presumably through mPTP opening) and cell damage. Representative Western blots of the effector proteins (RISK and SAFE pathways, together with cleaved caspase 3) are provided in Figure 6.

**Figure 7:**
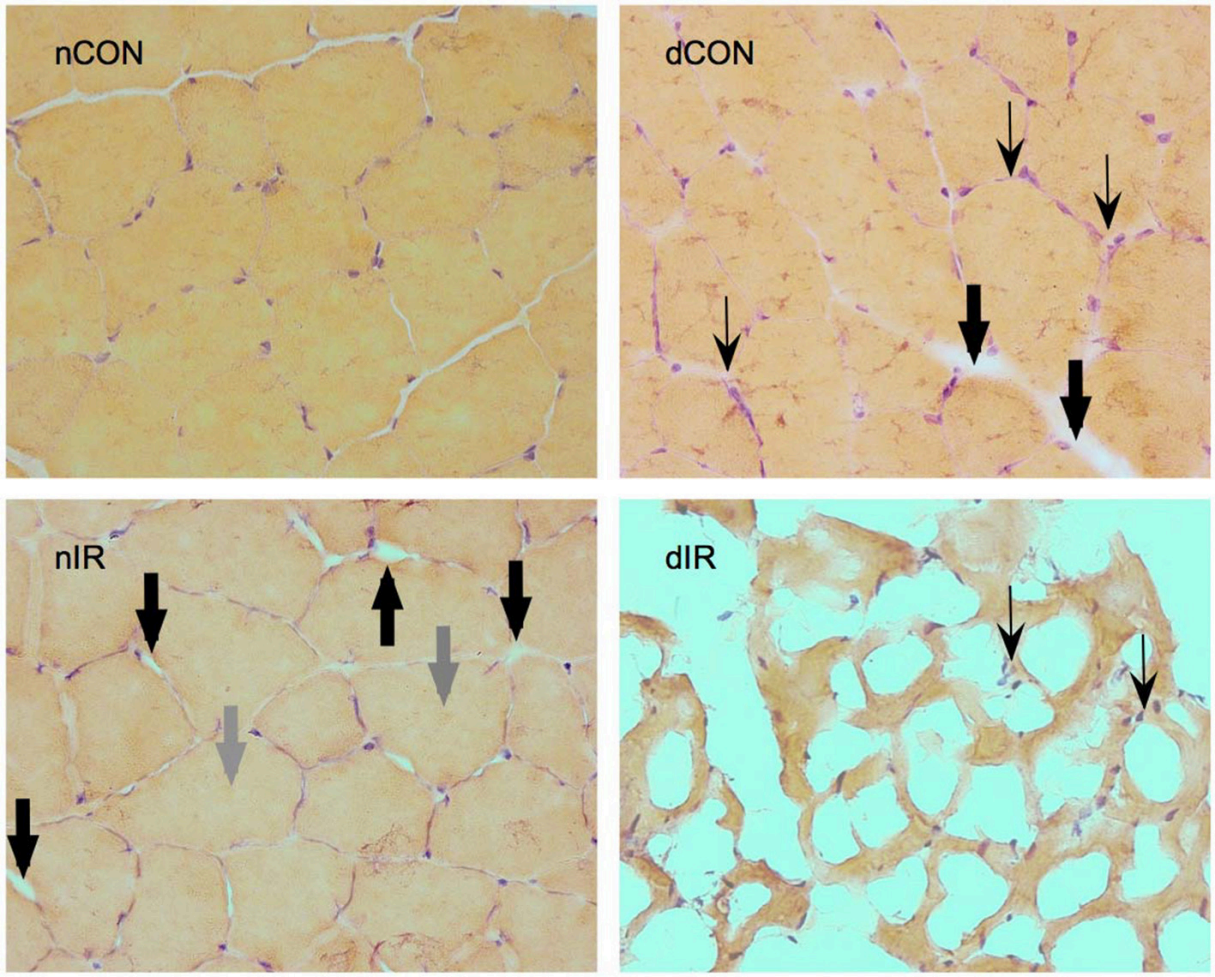
Hematoxylin-eosin staining of gastrocnemius muscles from sham-operated, vehicle-treated (nCON) and streptozotocin-treated rats (dCON), and after ischemia reperfusion in vehicle-treated (nIR) and streptozotocin-treated rats (dIR). Magnification ×400. Compared to nCON, gastrocnemius muscles from dCON rats showed reduced muscle fiber diameter, increased number of inflammatory cells invading the intercellular space (thin black, swept arrowheads) and intercellular edema (large black, solid arrowheads). After IR, gastrocnemius muscles from the nIR group displayed both increased intercellular and intracellular edema (large gray, solid arrowheads). After IR, gastrocnemius from the dIR group showed totally distorted anatomy with extensive necrosis and cell lysis surrounded by clusters of inflammatory cells (thin black, swept arrowheads).

Histological gastrocnemius samples from the diabetic-IR rats invariably showed totally distorted anatomy with extensive necrosis and cell lysis, compared to the non-diabetic-IR animals that presented much less severe cell injury (Figure 7).

## Discussion

The main finding of this study is that the impact of skeletal muscle IRI is worse in subjects with type 1 diabetes phenotype. Before ischemia, mitochondrial respiration and oxidative stress were more impaired in animals with diabetes than in those without, and these differences became more prominent after IR. Despite activation of one effector of the SAFE pathway, apoptosis and cell damage also appear to be more severe in diabetic subjects after IR. To the best of our knowledge, this is the first study that examined the combined effect of IR and diabetes on skeletal muscle.

Impaired skeletal muscle mitochondrial function and increased oxidative stress are the key elements of PAD-related IR injury (Tran et al., 2011; Guillot et al., 2014). Increased ROS production appears to precede (Guillot et al., 2014) and mediate mitochondrial dysfunction in skeletal muscles. In turn, dysfunctional mitochondria increase ROS production in a vicious cycle (Zorov et al., 2006; Chouchani et al., 2014). Type 1 and type 2 diabetes are also independently associated with increased oxidative stress and defective mitochondrial function, in both humans and experimental models (Kelley et al., 2002; Petersen et al., 2004; Karakelides et al., 2007; Bonnard et al., 2008; Anderson et al., 2009). Put in context, our results indicate that, after IR, diabetes further compromises mitochondrial function and oxidative stress in skeletal muscles, and suggest a cumulative or synergistic deleterious effect of both pathologies. A plausible mechanistic explanation could be that the diabetes-induced compromised mitochondrial function may be predisposing the limb muscles to respond poorly to IRI.

In the same line of evidence, Ryan et al., investigating the impact of type 2 diabetes on mice with acute limb ischemia, also reported exacerbation of skeletal muscle mitochondriopathy, oxidative stress and necrosis in diabetic animals. Although they did not extend their observations to the reperfusion period, they reported a reversal of these disturbances, by overexpression of catalase-mediated antioxidant defenses (Ryan et al., 2016). Bonnard et al. observed mitochondrial alterations that were similar in both type-1 and type-2 diabetic mice and associated with ROS production. Interestingly, the authors found that normalization of glycemia (by insulin) or antioxidant treatment restored mitochondrial integrity and reversed the proapoptotic process in the type-1 diabetic animals (Bonnard et al., 2008). Other studies, focusing on diabetic myocardium, have also shown that diabetes was associated with increased susceptibility to IRI (Whittington et al., 2012). Most of these studies suggested that the hyperglycemia-induced elevated levels of ROS and depletion of antioxidants were the key players in diabetic IRI (Ceriello et al., 2000; Nishikawa et al., 2000; Song et al., 2007).

Although present in skeletal muscles (Sandri, 2008), RISK and SAFE pathways activation has not been previously examined before in skeletal muscle IR. In this study, reduced Akt activation in diabetic animals indicates that the RISK pathway is not activated, and is consistent with the low inhibition of GSK-3β. GSK-3β is the downstream point of convergence of the RISK pathway and of several other prosurvival pathways and when phosphorylated (and thus inhibited) prevents mPTP opening (Juhaszova et al., 2004). Therefore, the lower levels of inactive GSK-3β in diabetic rats after IR may suggest failure of RISK and other protective pathways, ultimately resulting in mPTP opening, apoptosis and cell death. Consistently, downstream to GSK-3β, the proapoptotic cleaved caspase-3 levels were several times higher in diabetic rats. Several cardiac studies in diabetes also reported that hyperglycemia, insulin deficiency and insulin resistance were associated with loss of the conditioning-mediated cardioprotection and that this effect was driven by impaired signaling to Akt and GSK3β of the RISK pathway (Tsang et al., 2005; Gross et al., 2007; Song et al., 2007). In cardiomyocytes, upon IR, Akt activation appeared to be maximal after 10 min, remained elevated until 60 min of reperfusion, and returned toward basal levels by 2 h (Mockridge et al., 2000). Notwithstanding the lack of RISK activation at 2 h of reperfusion in our experiment, activation of RISK at an earlier timepoint cannot be excluded.

In contrast to RISK, SAFE pathway appeared to be activated in our diabetic animals after IR, as we observed an increased activation of STAT3. As a part of the SAFE pathway, STAT3 is involved in ischemic conditioning. Through its localization in the mitochondria of several organs, STAT3 modulates mitochondrial respiration and attenuates ROS production, mPTP opening and apoptosis (Wegrzyn et al., 2009). SAFE activation in diabetic animals should have been protective and yet, we found much higher cleaved caspase-3 levels in this group. These seemingly contradictory results may have several explanations. SAFE and RISK activation protects ischemic myocardium when triggered acutely, at the very first minutes of reperfusion, by specific conditioning maneuvers or pharmacological agents (Rossello and Yellon, 2017). In our study, STAT3 activation was spontaneous and hence, may not be sufficient or not activated early enough, to completely counteract the brunt of the increased oxidative stress. While spontaneous STAT3 activation has been shown to occur after myocardial ischemia and was further increased by reperfusion, it failed to reduce the infarct size (Hausenloy et al., 2011). Interestingly, STAT3 activation, despite its acute protective myocardial effects after IR, has also been strongly implied in atrophying skeletal muscle in experimental models of cancer cachexia or renal failure (Piccirillo and Giavazzi, 2015). Contrary to many chronic conditions, STAT3 activation usually triggers protective mechanisms in acute situations (like IR), probably accounting for the widespread “SAFE” label. In some situations, like in our study, one should probably not use the “SAFE” acronym, because STAT3 is not protective.

In our study, activated cleaved caspase-3 was several times higher in diabetic than in the non-diabetic subjects after IR, suggesting a strong trend toward apoptosis and cell death. Our histological findings, although not formally quantified, strongly corroborated this assertion. Chowdhry et al. reported comparable results in diabetic human myocardium. They demonstrated that diabetes increased both necrosis and apoptosis after IR, and that this effect was mediated by caspase-3 (Chowdhry et al., 2007). Other studies have also shown that increased activation of caspase-3 increased myocardial infarct size and that inhibiting caspases at the time of reperfusion, limited the infarct size (Mocanu et al., 2000; Condorelli et al., 2001). In skeletal muscles, caspases also mediate apoptosis after IR and trigger accelerated proteolysis in catabolic conditions (Du et al., 2004; Tran et al., 2012).

Several limitations of this study need to be acknowledged. Although the general limitations of the preclinical models may also apply to our study, we used a well-characterized pharmacological model of type 1 diabetes. Our data are descriptive and do not test specific mechanisms. That these pathways and mitochondrial function are altered in specific ways, simply make a series of observations and causality cannot be established. RISK and SAFE in skeletal muscle are investigated for the first time in this IR study and the analyses were performed at a single timepoint, after 3 h of ischemia and 2 h of reperfusion. In this regard, further studies are needed to determine the kinetics and the mechanisms of the different effectors. Executioner caspase-3 alone, although strongly inferring apoptosis and cell damage, cannot formally be interpreted as evidence of ultimate cell death. However, the histological findings seem to confirm a more extensive cell damage in the diabetic group.

In conclusion, our experimental data demonstrate that mitochondrial dysfunction, oxidative stress and apoptosis are enhanced in type 1 diabetes, after lower-limb IR. The so-called RISK and SAFE protective pathways appeared not to be activated in diabetic animals after IR, despite STAT 3 enhancement, potentially explaining the increased susceptibility to apoptosis and cell damage. These disturbances may contribute toward the high failure rate of revascularization in diabetic subjects with PAD. More studies are needed to further elucidate the mechanisms of IR mitochondriopathy in diabetes, determine the impact of specific anti-diabetic treatments on mitochondrial IR dysfunction and clarify the kinetics and the role of the protective pathways. Therapeutic approaches targeting mitochondria may reduce lower-limb IR damage and improve local and general prognosis in patients with PAD.

## Author contributions

Conception or design of the work JP, AL, JB, A-LC, GL, BG. Acquisition, analysis JP, CA, AL, JB, A-LC, AM, MS, VW, PD, GL, DM, BG. Interpretation of data for the work JP, CA, AL, JB, A-LC, AM, MS, VW, PD, GL, DM, BG. Drafting or revising the work: JP, CA, AL, AM, GL, BG. Final approval JP, CA, AL, JB, A-LC, AM, MS, VW, PD, GL, DM, BG. Agreement to be accountable of all aspects of the work JP, CA, AL, JB, A-LC, AM, MS, VW, PD, GL, DM, BG.

### Conflict of interest statement

The authors declare that the research was conducted in the absence of any commercial or financial relationships that could be construed as a potential conflict of interest.

## Abbreviations

Akt: protein kinase B
DHE: dihydroethidium
GAPDH: glyceraldehyde 3-phosphate dehydrogenase
GSK-3β: glycogen synthase kinase 3β
IR: ischemia-reperfusion
IRI: ischemia-reperfusion injury
mPTP: mitochondrial permeability transition pore
PAD: peripheral arterial disease
RISK: reperfusion injury salvage kinases
ROS: reactive oxygen species
SAFE: survivor activating factor enhancement
SOD2: mitochondrial manganese superoxide dismutase
STAT3: signal transducer and activator of transcription 3
TMPD, N, N, N': N'-tetramethyl-p-phenylenediamine dihydrochloride.

## References

[B1] Anderson E. J. Lustig M. E. Boyle K. E. Woodlief T. L. Kane D. A. Lin C. T.. (2009). Mitochondrial H2O2 emission and cellular redox state link excess fat intake to insulin resistance in both rodents and humans. J. Clin. Invest. 119, 573–581. 10.1172/JCI37048 19188683 PMC2648700

[B2] Bonnard C. Durand A. Peyrol S. Chanseaume E. Chauvin M. A. Morio B.. (2008). Mitochondrial dysfunction results from oxidative stress in the skeletal muscle of diet-induced insulin-resistant mice. J. Clin. Invest. 118, 789–800. 10.1172/JCI32601 18188455 PMC2176186

[B3] Ceriello A. Morocutti A. Mercuri F. Quagliaro L. Moro M. Damante G.. (2000). Defective intracellular antioxidant enzyme production in type 1 diabetic patients with nephropathy. Diabetes 49, 2170–2177. 10.2337/diabetes.49.12.2170 11118022

[B4] Charles A. L. Guilbert A. S. Bouitbir J. Goette-Di Marco P. Enache I. Zoll J.. (2011). Effect of postconditioning on mitochondrial dysfunction in experimental aortic cross-clamping. Br. J. Surg. 98, 511–516. 10.1002/bjs.7384 21259232

[B5] Charles A. L. Guilbert A.-S. Guillot M. Talha S. Lejay A. Meyer A.. (2017). Muscles susceptibility to ischemia-reperfusion injuries depends on fiber type specific antioxidant level. Front. Physiol. 8:52. 10.3389/fphys.2017.00052 28220081 PMC5292410

[B6] Chouchani E. T. Pell V. R. Gaude E. Aksentijević D. Sundier S. Y. Robb E. L.. (2014). Ischaemic accumulation of succinate controls reperfusion injury through mitochondrial ROS. Nature 515, 431–435. 10.1038/nature13909 25383517 PMC4255242

[B7] Chowdhry M. F. Vohra H. A. Galiñanes M. (2007). Diabetes increases apoptosis and necrosis in both ischemic and nonischemic human myocardium: role of caspases and poly-adenosine diphosphate-ribose polymerase. J. Thorac. Cardiovasc. Surg. 134, 124–131, 131.e121–123. 10.1016/j.jtcvs.2006.12.059 17599497

[B8] Condorelli G. Roncarati R. Ross J. Jr. Pisani A. Stassi G. Todaro M.. (2001). Heart-targeted overexpression of caspase3 in mice increases infarct size and depresses cardiac function. Proc. Natl. Acad. Sci. U.S.A. 98, 9977–9982. 10.1073/pnas.161120198 11493678 PMC55563

[B9] DeRubertis B. G. Pierce M. Ryer E. J. Trocciola S. Kent K. C. Faries P. L. (2008). Reduced primary patency rate in diabetic patients after percutaneous intervention results from more frequent presentation with limb-threatening ischemia. J. Vasc. Surg. 47, 101–108. 10.1016/j.jvs.2007.09.018 18178459

[B10] Drenger B. Ostrovsky I. A. Barak M. Nechemia-Arbely Y. Ziv E. Axelrod J. H. (2011). Diabetes blockade of sevoflurane postconditioning is not restored by insulin in the rat heart: phosphorylated signal transducer and activator of transcription 3- and phosphatidylinositol 3-kinase-mediated inhibition. Anesthesiology 114, 1364–1372. 10.1097/ALN.0b013e31820efafd 21368653

[B11] Du J. Wang X. Miereles C. Bailey J. L. Debigare R. Zheng B.. (2004). Activation of caspase-3 is an initial step triggering accelerated muscle proteolysis in catabolic conditions. J. Clin. Invest. 113, 115–123. 10.1172/JCI18330 14702115 PMC300763

[B12] Gross E. R. Hsu A. K. Gross G. J. (2007). Diabetes abolishes morphine-induced cardioprotection via multiple pathways upstream of glycogen synthase kinase-3beta. Diabetes 56, 127–136. 10.2337/db06-0907 17192474

[B13] Guillot M. Charles A. L. Chamaraux-Tran T. N. Bouitbir J. Meyer A. Zoll J.. (2014). Oxidative stress precedes skeletal muscle mitochondrial dysfunction during experimental aortic cross-clamping but is not associated with early lung, heart, brain, liver, or kidney mitochondrial impairment. J. Vasc. Surg. 60, 1043–1051 e1045. 10.1016/j.jvs.2013.07.100 24095040

[B14] Hausenloy D. J. Lecour S. Yellon D. M. (2011). Reperfusion injury salvage kinase and survivor activating factor enhancement prosurvival signaling pathways in ischemic postconditioning: two sides of the same coin. Antioxid. Redox Signal. 14, 893–907. 10.1089/ars.2010.3360 20615076

[B15] Herlein J. A. Fink B. D. Henry D. M. Yorek M. A. Teesch L. M. Sivitz W. I. (2011). Mitochondrial superoxide and coenzyme Q in insulin-deficient rats: increased electron leak. Am. J. Physiol. Regul. Integr. Comp. Physiol. 301, R1616–R1624. 10.1152/ajpregu.00395.2011 21940403 PMC3233854

[B16] Jude E. B. Oyibo S. O. Chalmers N. Boulton A. J. (2001). Peripheral arterial disease in diabetic and nondiabetic patients: a comparison of severity and outcome. Diabetes Care 24, 1433–1437. 10.2337/diacare.24.8.1433 11473082

[B17] Juhaszova M. Zorov D. B. Kim S. H. Pepe S. Fu Q. Fishbein K. W.. (2004). Glycogen synthase kinase-3beta mediates convergence of protection signaling to inhibit the mitochondrial permeability transition pore. J. Clin. Invest. 113, 1535–1549. 10.1172/JCI19906 15173880 PMC419483

[B18] Karakelides H. Asmann Y. W. Bigelow M. L. Short K. R. Dhatariya K. Coenen-Schimke J.. (2007). Effect of insulin deprivation on muscle mitochondrial ATP production and gene transcript levels in type 1 diabetic subjects. Diabetes 56, 2683–2689. 10.2337/db07-0378 17660267

[B19] Kelley D. E. He J. Menshikova E. V. Ritov V. B. (2002). Dysfunction of mitochondria in human skeletal muscle in type 2 diabetes. Diabetes 51, 2944–2950. 10.2337/diabetes.51.10.2944 12351431

[B20] Lecour S. (2009). Multiple protective pathways against reperfusion injury: a SAFE path without Aktion? J. Mol. Cell. Cardiol. 46, 607–609. 10.1016/j.yjmcc.2009.01.003 19318238

[B21] Lejay A. Laverny G. Paradis S. Schlagowski A. I. Charles A. L. Singh F.. (2017). Moderate exercise allows for shorter recovery time in critical limb ischemia. Front. Physiol. 8:523. 10.3389/fphys.2017.00523 28790926 PMC5524729

[B22] Lejay A. Meyer A. Schlagowski A. I. Charles A. L. Singh F. Bouitbir J.. (2014). Mitochondria: mitochondrial participation in ischemia-reperfusion injury in skeletal muscle. Int. J. Biochem. Cell Biol. 50, 101–105. 10.1016/j.biocel.2014.02.013 24582887

[B23] Li C. Jackson R. M. (2002). Reactive species mechanisms of cellular hypoxia-reoxygenation injury. Am. J. Physiol. Cell Physiol. 282, C227–C241. 10.1152/ajpcell.00112.2001 11788333

[B24] Makris K. I. Nella A. A. Zhu Z. Swanson S. A. Casale G. P. Gutti T. L.. (2007). Mitochondriopathy of peripheral arterial disease. Vascular 15, 336–343. 10.2310/6670.2007.00054 18053417

[B25] Malmstedt J. Leander K. Wahlberg E. Karlstrom L. Alfredsson L. Swedenborg J. (2008). Outcome after leg bypass surgery for critical limb ischemia is poor in patients with diabetes: a population-based cohort study. Diabetes Care 31, 887–892. 10.2337/dc07-2424 18268064

[B26] Mansour Z. Bouitbir J. Charles A. L. Talha S. Kindo M. Pottecher J.. (2012). Remote and local ischemic preconditioning equivalently protects rat skeletal muscle mitochondrial function during experimental aortic cross-clamping. J. Vasc. Surg. 55, 497–505 e491. 10.1016/j.jvs.2011.07.084 22056287

[B27] Mocanu M. M. Baxter G. F. Yellon D. M. (2000). Caspase inhibition and limitation of myocardial infarct size: protection against lethal reperfusion injury. Br. J. Pharmacol. 130, 197–200. 10.1038/sj.bjp.0703336 10807653 PMC1572087

[B28] Mockridge J. W. Marber M. S. Heads R. J. (2000). Activation of Akt during simulated ischemia/reperfusion in cardiac myocytes. Biochem. Biophys. Res. Commun. 270, 947–952. 10.1006/bbrc.2000.2522 10772931

[B29] Nishikawa T. Edelstein D. Du X. L. Yamagishi S. Matsumura T. Kaneda Y.. (2000). Normalizing mitochondrial superoxide production blocks three pathways of hyperglycaemic damage. Nature 404, 787–790. 10.1038/35008121 10783895

[B30] Paradis S. Charles A. L. Meyer A. Lejay A. Scholey J. W. Chakfé N.. (2016). Chronology of mitochondrial and cellular events during skeletal muscle ischemia-reperfusion. Am. J. Physiol,. Cell Physiol. 310, C968–C982. 10.1152/ajpcell.00356.2015 27076618 PMC4935201

[B31] Petersen K. F. Dufour S. Befroy D. Garcia R. Shulman G. I. (2004). Impaired mitochondrial activity in the insulin-resistant offspring of patients with type 2 diabetes. N. Engl. J. Med. 350, 664–671. 10.1056/NEJMoa031314 14960743 PMC2995502

[B32] Piccirillo R. Giavazzi R. (2015). Inactivating STAT3: bad for tumor, good for muscle. Cell Cycle 14, 939–940. 10.1080/15384101.2015.1010979 25668448 PMC4613659

[B33] Pipinos I. I. Judge A. R. Zhu Z. Selsby J. T. Swanson S. A. Johanning J. M.. (2006). Mitochondrial defects and oxidative damage in patients with peripheral arterial disease. Free Radic. Biol. Med. 41, 262–269. 10.1016/j.freeradbiomed.2006.04.003 16814106

[B34] Pottecher J. Guillot M. Belaidi E. Charles A. L. Lejay A. Gharib A.. (2013). Cyclosporine A normalizes mitochondrial coupling, reactive oxygen species production, and inflammation and partially restores skeletal muscle maximal oxidative capacity in experimental aortic cross-clamping. J. Vasc. Surg. 57, 1100–1108 e1102. 10.1016/j.jvs.2012.09.020 23332985

[B35] Rossello X. Yellon D. M. (2017). The RISK pathway and beyond. Basic Res. Cardiol. 113:2. 10.1007/s00395-017-0662-x 29143177 PMC5688212

[B36] Ryan T. E. Schmidt C. A. Green T. D. Spangenburg E. E. Neufer P. D. McClung J. M. (2016). Targeted expression of catalase to mitochondria protects against ischemic myopathy in high-fat diet-fed mice. Diabetes 65, 2553–2568. 10.2337/db16-0387 27284110 PMC5001179

[B37] Sandri M. (2008). Signaling in muscle atrophy and hypertrophy. Physiology (Bethesda) 23, 160–170. 10.1152/physiol.00041.2007 18556469

[B38] Sheetz M. J. King G. L. (2002). Molecular understanding of hyperglycemia's adverse effects for diabetic complications. JAMA 288, 2579–2588. 10.1001/jama.288.20.2579 12444865

[B39] Song P. Wu Y. Xu J. Xie Z. Dong Y. Zhang M.. (2007). Reactive nitrogen species induced by hyperglycemia suppresses Akt signaling and triggers apoptosis by upregulating phosphatase PTEN (phosphatase and tensin homologue deleted on chromosome 10) in an LKB1-dependent manner. Circulation 116, 1585–1595. 10.1161/CIRCULATIONAHA.107.716498 17875968

[B40] Talha S. Bouitbir J. Charles A. L. Zoll J. Goette-Di Marco P. Meziani F.. (2013). Pretreatment with brain natriuretic peptide reduces skeletal muscle mitochondrial dysfunction and oxidative stress after ischemia-reperfusion. J. Appl. Physiol. (1985) 114, 172–179. 10.1152/japplphysiol.00239.2012 23104692

[B41] Tran T. P. Tu H. Liu J. Muelleman R. L. Li Y. L. (2012). Mitochondria-derived superoxide links to tourniquet-induced apoptosis in mouse skeletal muscle. PLoS ONE 7:e43410. 10.1371/journal.pone.0043410 22912870 PMC3422247

[B42] Tran T. P. Tu H. Pipinos I. I. Muelleman R. L. Albadawi H. Li Y. L. (2011). Tourniquet-induced acute ischemia-reperfusion injury in mouse skeletal muscles: Involvement of superoxide. Eur. J. Pharmacol. 650, 328–334. 10.1016/j.ejphar.2010.10.037 21036124 PMC3008320

[B43] Tsang A. Hausenloy D. J. Mocanu M. M. Carr R. D. Yellon D. M. (2005). Preconditioning the diabetic heart: the importance of Akt phosphorylation. Diabetes 54, 2360–2364. 10.2337/diabetes.54.8.2360 16046302

[B44] Wegrzyn J. Potla R. Chwae Y. J. Sepuri N. B. Zhang Q. Koeck T.. (2009). Function of mitochondrial Stat3 in cellular respiration. Science 323, 793–797. 10.1126/science.1164551 19131594 PMC2758306

[B45] Whittington H. J. Babu G. G. Mocanu M. M. Yellon D. M. Hausenloy D. J. (2012). The diabetic heart: too sweet for its own good? Cardiol. Res. Pract. 2012:845698. 10.1155/2012/845698 22462028 PMC3296224

[B46] Zorov D. B. Juhaszova M. Sollott S. J. (2006). Mitochondrial ROS-induced ROS release: an update and review. Biochim. Biophys. Acta 1757, 509–517. 10.1016/j.bbabio.2006.04.029 16829228

